# Editorial for the Special Issue on Physics in Micro/Nano Devices: From Fundamental to Application

**DOI:** 10.3390/mi14081571

**Published:** 2023-08-08

**Authors:** Ridong Wang, Zhihua Pu

**Affiliations:** State Key Laboratory of Precision Measuring Technology and Instruments, Tianjin University, Tianjin 300072, China

With the continuous miniaturization of micro/nano devices, it is of great importance to study the physics of these devices, both for fundamental and practical research. The scope of this Special Issue is very wide, including light scattering, bubble behaviors, microfluidics, electrochemical analysis, surface plasmon resonance (SPR), surface acoustic waves (SAWs), etc. Of the 14 papers published in the Special Issue, 2 are review articles and 12 are original research papers, which include 6 fundamental research papers and 6 practical research papers. The two review articles, which focus on SAW filters [1] and modular microfluidics [2], present in-depth discussions on the current status and future prospects of these two popular topics.

Among the six fundamental research papers, two are related to light scattering from nanostructures. Wan et al. proposed an efficient numerical method based on MoM and a hierarchical matrix algorithm to predict light scattering from plasmonic nanoarrays in multiple directions [3]. Liu et al. proposed an accelerated algorithm that can efficiently calculate the light scattering of a single metal nanoparticle [4]. When it comes to nanoparticles, as the impurity nanoparticles affect the yield rate of semiconductor production, Jang et al. conducted a numerical study of ellipsoidal nanoparticles under high vacuum using the direct simulation Monte Carlo method [5]. The motion of bubbles in an ultrasonic field is also a fundamental physical mechanism in most applications of acoustic cavitation. Wu et al. examined the influence of liquids’ surface tension on single micro-bubbles’ motion in an ultrasonic field, which is helpful to understand the mechanism of surfactants in promoting acoustic cavitation in numerous application fields [6]. In micro/nano systems, continuum description of flows is of great importance. However, a sound theoretical ground unifying effects, such as slip at walls, surface diffusion, and Knudsen diffusion, is still lacking. Tomy et al. suggested the derivation of model boundary conditions that may systematically justify various diffusion processes occurring in micro/nano-flows where the classical continuum model breaks down [7]. In thermoelectric devices, Zhao et al. developed a variable-range hopping (VRH) theory-based model to reveal the thermoelectric properties in Gaussian disordered organic semiconductors, providing a good description of the relationship between the Seebeck coefficient and conductivity [8].

In the six practical research papers, different kinds of micro devices, including anemometers, electrode detectors, SPR aptasensors, electrochemical film powers, reconfigurable microfluidic platforms, and magnetically actuated platforms, are covered. Ye et al. investigated the effect of wind-induced vibration on the measurement range of a microcantilever anemometer for the first time, which can pave the way for the design and fabrication of wide-range mechanical anemometers [9]. Cai et al. proposed a new type of 3D electrode detector, named the Implanted Epi Silicon 3D Spherical Electrode Detector. Compared with the traditional silicon 3D electrode detectors, the full depletion voltage was greatly reduced, which showed high potential in photon science [10]. Zheng et al. designed an electrically inspired flexible electrochemical film power supply for long-term epidermal sensors for the first time, which can periodically provide electrical power for several hours after a short-time electrical activation and the electrical activation can be enabled by an integrated small film lithium-ion battery, which extends the service life of a lithium-ion battery 10-fold and suggests the application of small lithium-ion batteries for long-term epidermal sensors [11]. Hua et al. developed a fiber-based SPR biosensor decorated with DNA aptamers for the early diagnosis of cardiovascular disease. Integrated with a miniaturized spectral analysis device, the proposed sensor can be applied in the construction of portable instruments to provide point-of-care testing [12]. Lai et al. proposed a modular, reconfigurable microfluidic platform, which was inspired by the selflocking structure of the Rubik’s cube. This platform can realize the reliable interconnection and rapid rearrangement of microfluidic modules by simply rotating the faces of the microfluidic cube [13]. Lin et al. developed a platform for the magnetic manipulation of droplets containing magnetic beads and examined the washing behaviors of the droplets, which provided digital microfluidics for applications in point-of-care testing. The developed microchip will be of great benefit for genetic analysis and infectious disease detection in the future [14].

We hope that this Special Issue will offer readers a good overview of the different research areas related to micro/nano devices and attract more researchers devoted to this fast-growing area.
